# AFD Thermosensory Neurons Mediate Tactile-Dependent Locomotion Modulation in *C. elegans*

**DOI:** 10.1101/2025.02.19.639001

**Published:** 2025-02-24

**Authors:** Manuel Rosero, Jihong Bai

**Affiliations:** 1 Basic Sciences Division, Fred Hutchinson Cancer Center, Seattle, WA 98109; 2 Molecular and Cellular Biology Graduate Program, University of Washington, Seattle, WA 98109

## Abstract

Sensory neurons drive animal behaviors by detecting environmental stimuli and relaying information to downstream circuits. Beyond their primary roles in sensing, these neurons often form additional synaptic connections outside their main sensory modality, suggesting broader contributions to behavior modulation. Here, we uncover an unexpected role for the thermosensory neuron AFD in coupling tactile experience to locomotion modulation in *Caenorhabditis elegans*. We show that while AFD employs cGMP signaling for both thermotaxis and tactile-dependent modulation, the specific molecular components of the cGMP pathway differ between these two processes. Interestingly, disrupting the dendritic sensory apparatus of AFD, which is essential for thermotaxis, does not impair tactile-based locomotion modulation, indicating that AFD can mediate tactile-dependent behavior independently of its thermosensory apparatus. In contrast, ablating the AFD neuron eliminates tactile-dependent modulation, pointing to an essential role for AFD itself, rather than its sensory dendritic endings. Further, we find tactile-dependent modulation requires the AIB interneuron, which connects AFD to touch circuits via electrical synapses. Removing innexins expressed in AFD and AIB abolishes this modulation, while re-establishing AFD-AIB connections with engineered electrical synapses restores it. Collectively, these findings uncover a previously unrecognized function of AFD beyond thermosensation, highlighting its influence on context-dependent neuroplasticity and behavioral modulation through broader circuit connectivity.

## INTRODUCTION

Adaptive behavioral responses require animals to interpret sensory signals in the context of prior experiences, enabling effective responses to changing environmental conditions. For example, rodents associate contextual cues, such as odors and colors, with rewards or punishments, leading to context-dependent adjustments in approach or avoidance behaviors [1,2]. Disruptions in the biological processes underlying this plasticity, as seen in neurological and psychiatric disorders, often result in impaired behavior modulation based on prior sensory experiences [3–7]. Recognizing the critical role of context-dependent behavioral modulation for survival and well-being, ongoing research focuses on uncovering the neuroplastic mechanisms responsible for these adaptive processes.

Studies across diverse organisms reveal that animals, regardless of nervous system complexity, integrate prior sensory experiences to shape behavior [8–15]. In the nematode *C. elegans*, which has only 302 neurons, locomotion strategies are modified by prior exposure to stimuli such as odors, touch, light, and starvation, demonstrating robust adaptive behaviors [16–23]. The wide range of adaptive behaviors, combined with the well-mapped *C. elegans* nervous system and abundant genetic tools, have empowered the discovery of fundamental circuit mechanisms and molecular pathways underlying context-dependent behavioral modulation [24–28].

The *C. elegans* sensory system comprises 60 ciliated sensory neurons specialized in detecting chemical, thermal, mechanical, and olfactory stimuli [29,30]. Each sensory neuron has a dendritic apparatus that converts specific environmental signals into neuronal activity, which drives signal flow through downstream networks of interneurons and motor neurons, ultimately shaping behavior [31–33]. Among these, AFD, a pair of bilaterally symmetric left-right neurons, serves as the primary thermosensory neurons required for thermotaxis (movement toward the a preferred temperature) [34]. The dendritic endings of AFD, enriched with microvilli, serve as the primary site for thermal detection. Even when detached from the cell body, these dendritic endings retain calcium responsiveness to temperature stimuli [35]. Moreover, genetically disrupting these sensory endings abolishes the Ca^2+^ response in AFD cell bodies to temperature changes and eliminates thermotaxis, underscoring their essential role in thermal sensing [36–38].

The second messenger cyclic guanosine monophosphate (cGMP) plays a central role in modulating AFD thermosensory activity. Temperature changes in AFD neurons trigger intracellular Ca^2+^ dynamics, with Ca^2+^ levels rising during warming and falling during cooling [35,39,40]. This response relies on cGMP synthesis mediated by the guanylyl cyclases (GCs) *gcy-8*, *gcy-18*, and *gcy-23*, which have overlapping but distinct roles. Specifically, *gcy-8* is essential for generating thermally evoked Ca^2+^ responses in AFD [41,42], whereas *gcy-18* and *gcy-23* act synergistically to sustain temperature sensing [41,43]. The cGMP produced by these cyclases activates cyclic nucleotide-gated (CNG) channels, comprised of alpha-subunits TAX-4 and beta-subunits TAX-2, resulting in increases in intracellular Ca^2+^. Mutations in either *tax-4* or *tax-2* abolish thermally evoked Ca^2+^ responses in AFD and disrupt thermotaxis, underscoring the importance of cGMP signaling for AFD-mediated thermosensation [40].

AFD transmits thermal signals to several downstream interneurons, including AIY, AIZ, and AIB, which process this information to guide temperature-dependent behaviors. Thermotaxis in *C. elegans* encompasses three categories: movement toward warmer temperatures (thermophilic), movement toward cooler temperatures (cryophilic), and movement within a preferred temperature range (isothermal) [44,45]. Each behavior type depends on distinct AFD synaptic partners: AIY is critical for thermophilic and isothermal behaviors [46,47]; AIZ is specific for cryophilic behavior [48–50]; and AIB also supports thermophilic behavior, though its role is less critical than that of AIY [51]. These findings demonstrate the functional specificity of synaptic partners of AFD neurons in shaping distinct temperature-dependent behaviors.

In this study, we demonstrate that AFD plays a novel role in tactile-mediated, context-dependent locomotion modulation, thereby expanding its function beyond temperature sensing. The mechanisms underlying this role differ markedly from those involved in thermosensation. First, context-dependent modulation requires distinct cGMP signaling components. Second, while the microvilli-rich sensory endings of AFD are essential for thermosensation, they are dispensable for context-dependent modulation. Third, this modulation depends on mechanosensory neurons and on electrical synapses between AFD and the AIB interneuron, which connects to mechanosensory circuits. Finally, disruption of *inx-7* and *inx-10* genes, encoding gap junction protein innexins in AFD and AIB, impairs context-dependent locomotion modulation. Remarkably, restoring AFD–AIB connectivity via engineered Cx36 electrical synapses rescues this function. Collectively, our findings uncover an unconventional mechanism by which AFD mediates behavior modulation based on tactile experience.

## RESULTS

### *C. elegans* displays distinct locomotion in identical environments after exploring different physical structures.

**Figure 1.**
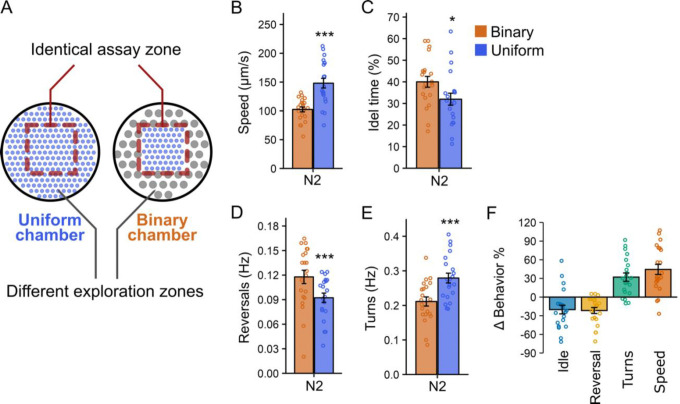
*C. elegans* displays distinct locomotion in identical environments after exploring different surrounding areas. (A) Schematic of two microfluidic chamber designs—uniform and binary—used for behavioral assays. Both chambers feature an identical assay area (red dashed-line boxes) where locomotion was analyzed, while the exploration zones contain distinct PDMS pillar patterns for worm exploration. (B–E) Bar graphs showing locomotion properties of wild type N2 worms in the assay zones of both chamber types after 1 hour of exploration. Data from binary chambers are shown in orange and from uniform chambers in blue. Shown are (B) locomotion speed, (C) percentage of time spent idle, (D) reversal frequency, and (E) turning frequency. Error bars represent standard error of the mean (SEM); each data point represents the mean behavior value of worms within a chamber. (F) Percent changes in locomotion properties, calculated from data in panels B–E and normalized to the mean value obtained from binary chambers (see Methods), are presented as mean ± SEM. Statistical significance was determined using an unpaired Student’s t-test (***: p < 0.001, *: p < 0.05).

To investigate the impact of prior experience on *C. elegans* behavior, we first allowed worms to explore chambers with distinct pillar designs (exploration zone, Fig. 1A) before recording their locomotion in identical assay zones (red dashed-line boxes, Fig. 1A). We hypothesized that if behavior were solely driven by immediate sensory input, worms in identical assay zones would exhibit similar locomotion. Conversely, if prior experience, such as exposure to different physical features in the exploration zones, influences behavior, then worms should display distinct locomotor patterns even in the identical assay zones. To test these hypotheses, we employed two types of microfluidic chambers: uniform and binary (Fig. 1A). Both chamber types include an identical assay zone. Once worms enter this zone, their immediate environment is the same regardless of the chamber. However, before reaching the assay zone, worms explore surrounding areas with pillar designs that differ in size and spacing. This design allowed us to assess how prior exposure to different physical contexts impacts locomotion in an otherwise identical setting.

Worms were allowed to explore the chambers for 60 minutes before their behavior was recorded in the assay zones. We found that worms in the uniform chamber assay zone exhibited locomotor behavior that differed from that of worms in the binary chamber assay zone (Fig. 1B–F). Specifically, in the uniform chamber assay zone, worms moved approximately 40% faster (Fig. 1B, F), spent about 20% less time idle (Fig. 1C, F), reversed direction around 20% less frequently (Fig. 1D, F), and turned about 30% more often (Fig. 1E, F) than worms in the binary chamber assay zone. These findings demonstrate that locomotion adjustments are shaped by past sensory experience rather than solely by immediate environmental cues. That worms exhibited different behaviors in identical assay zones highlights the flexible, context-dependent nature of *C. elegans* behavior.

### Guanylyl cyclase gene *gcy-18* is required for context-dependent locomotion modulation in chambers.

**Figure 2.**
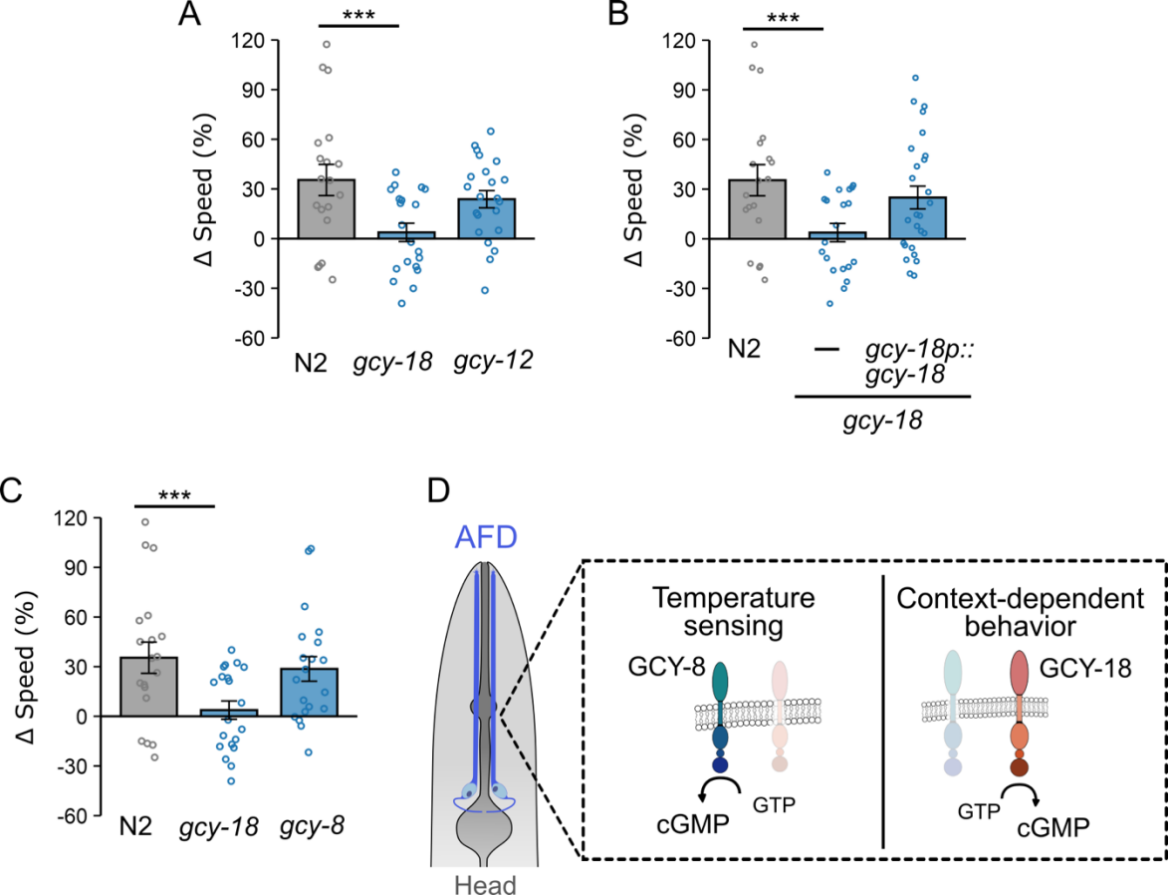
Guanylate cyclase gene *gcy-18* functions in AFD to mediate context-dependent locomotion modulation. (A) Speed differences (Δspeed) between uniform and binary chambers of wild type N2, *gcy-18* and *gcy-12* mutant worms. ΔSpeed was calculated as the percent change in locomotion rates in uniform chambers, relative to the mean of values obtained in binary chambers (see Methods). Mutant worms lacking *gcy-18* failed to demonstrate context-dependent adjustments in locomotion speed, as indicated by a low Δspeed value, whereas *gcy-12* mutants behaved similarly to wild type worms. (B) A single-copy transgene expressing *gcy-18* cDNA under the control of the *gcy-8* promoter in AFD restores context-dependent locomotion modulation. (C) Disruption of *gcy-8* does not impair context-dependent locomotion modulation. (D) Schematic illustrating the distinct roles of guanylate cyclase genes in AFD sensory neurons: *gcy-8* is required for thermosensation but not for context-dependent locomotion modulation, whereas *gcy-18* is essential for context-dependent locomotion modulation and plays only a moderate role in thermosensation. Data are presented as mean ± SEM. Each data point represents the mean behavior of worms within a single chamber. Statistical significance was determined using one-way ANOVA followed by a Tukey–Kramer post hoc test (***: p < 0.001).

To identify mechanisms underlying context-dependent locomotion adjustments, we employed a previously reported preference assay that uses microfluidic arenas to assess the ability of worms to select a preferred space [52]. Because worms must adjust their locomotion based on their surroundings to remain within a preferred area, we hypothesized that mutations abolishing this preference would reveal key mechanisms underlying context-dependent adjustments in the uniform and binary chambers. Accordingly, we screened for mutant worms that failed to choose their preferred area, aiming to identify the genetic mechanisms mediating locomotion adjustments in response to different physical structures. Given the significant role of cGMP in neuronal plasticity and behavior modulation, we performed a small-scale genetic screen using loss-of-function mutants for 24 genes encoding guanylyl cyclase (GC), the enzyme responsible for cGMP synthesis. Using the preference assay, we identified two putative null mutants, *gcy-18(gk423024)* and *gcy-12(gk142661),* that failed to display a spatial preference (Supp Fig. 1). We then compared the locomotion properties of *gcy-18* and *gcy-12* mutants in both uniform and binary chambers. Our results showed that *gcy-18* mutants failed to execute context-dependent adjustments, displaying similar locomotion rates in both chamber types. Specifically, *gcy-18* mutants exhibited a significantly reduced Δspeed (3 ± 5%) compared to wild type (35 ± 7%, p<0.001, Fig. 2A). In contrast, *gcy-12* mutant worms exhibited normal speed adjustment, with Δspeed values comparable to wild type (Fig. 2A). This indicates that *gcy-12* is not required for the modulation of locomotion speed. In comparison, the failure of *gcy-18* mutants to adjust their speed suggests that *gcy-18* plays a critical role in context-dependent behavioral adjustment. Together, these results demonstrate that among the guanylyl cyclases, *gcy-18* is essential for the context-dependent modulation of locomotion.

To confirm that the defects observed in *gcy-18* mutants were not due to background artifacts, we introduced a single-copy transgene that drives *gcy-18* cDNA expression under the control of a *gcy-18* promoter into *gcy-18(gk423024)* mutants to test whether reintroducing a functional copy of *gcy-18* could rescue the behavioral defects. Indeed, expression of the transgene restored locomotion adjustments (Δspeed: 20 ± 6%) to levels closer to those observed in wild type animals (35 ± 7%, p = 0.4, Fig. 2B), confirming the crucial role of *gcy-18* in mediating these adjustments based on the experience of exploring physical environments. The identification of *gcy-18* was particularly intriguing, as its expression is restricted to a single pair of neurons, the thermosensory AFDs. Given that our assay chambers lacked thermal gradients, this finding suggests a novel role for *gcy-18* and AFD neurons in mediating locomotion adjustments in response to the exploration of surrounding physical structures.

To further investigate the role of AFD-specific guanylyl cyclases in context-dependent locomotion modulation, we examined *gcy-8*, which is essential for generating thermally evoked Ca^2+^ responses in AFD [41,42]. In thermal responses, disrupting *gcy-8* alone is sufficient to impair AFD function, whereas disruption of *gcy-18* alone does not [41,43], indicating the critical role of GCY-8 in AFD thermosensation. If *gcy-18* influenced behavioral adjustment in microfluidic chambers solely through its role in thermosensation, we would expect that a loss-of-function mutation in *gcy-8* would have a similar or even more disruptive impact on locomotion modulation. However, *gcy-8* mutant worms exhibit little change in their ability to adjust locomotion speed (Δspeed: 29 ± 7%), with Δspeed values similar to that observed in wild type worms (p = 0.96, Fig. 2C). These results indicate that while *gcy-8* is critical for thermosensation, its mutation does not affect context-dependent locomotion modulation, suggesting that independent cGMP signaling mechanisms govern AFD neuronal activity in thermosensation and context-dependent behavior in microfluidic chambers (Fig. 2D).

### Distinct CNG channels mediate context-dependent locomotion modulation and thermosensation.

**Figure 3.**
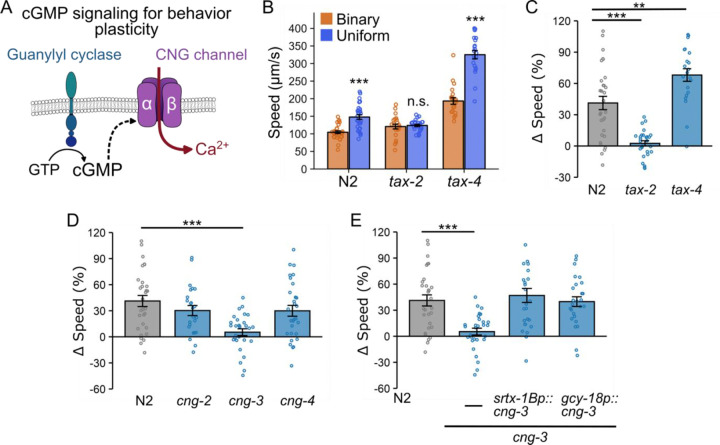
Cyclic nucleotide-gated (CNG) channel subunits TAX-2 and CNG-3 are required for locomotion modulation, but TAX-4 is not. (A) Schematic representation of CNG channel function. CNG channels are activated by cyclic nucleotides (such as cGMP) and mediate Ca^2+^ influx, thereby influencing neuronal activity and animal behavior. (B) Locomotion speed (μm/s) of N2, *tax-2*, and *tax-4* mutant worms in binary (orange) and uniform (blue) chambers. The *tax-2* mutants exhibit identical locomotion rates in both chamber types, whereas worms lacking *tax-4* display accelerated basal locomotion while still preserving context-dependent locomotion modulation. (C) Speed differences (Δspeed) for N2, *tax-2* mutant, and *tax-4* mutant worms. The *tax-2* mutants failed to modulate locomotion rates in a context-dependent manner, whereas *tax-4* mutations enhanced modulation. (D) Assessment of *cng-2*, *cng-3*, and *cng-4* roles in locomotion adjustments. The *cng-3* mutation abolishes locomotion modulation. (E) Single-copy transgenes expressing *cng-3* cDNA under two different AFD-specific promoters restore context-dependent locomotion adjustments. Data are presented as mean ± SEM. Each data point represents the mean behavior of worms within a single chamber. Statistical significance was determined using an unpaired Student’s t-test to compare speeds between uniform and binary chambers (panel B) and one-way ANOVA followed by a Tukey–Kramer post hoc test to compare Δspeed across strains (panels C-E) (*: p < 0.05; **: p < 0.01, and ***: p < 0.001).

The role of guanylyl cyclase in cGMP synthesis prompted us to investigate whether cyclic nucleotide-gated (CNG) channels are involved in context-dependent locomotion modulation. CNG channels, composed of α and β subunits, open upon binding cyclic nucleotides (*e.g.*, cGMP and cAMP), allowing Ca^2+^ influx [53–56] to control signal transduction and neural plasticity across species [57–64] (Fig. 3A). In *C. elegans*, mutations in either the *tax-4* or *tax-2* genes, which encode the α and β subunits of CNG channels respectively, impair thermotaxis behavior [47,65] and disrupt AFD neuron responses to temperature changes [40,66]. To determine their role in context-dependent locomotion modulation, we examined *tax-4(p678)* and *tax-2(p694)* mutant worms using our microfluidic chamber assay.

We found that *tax-*2 is essential for context-dependent locomotion modulation, as *tax-2* mutants did not exhibit differences in speed between chamber types (Fig. 3B, C). In contrast, *tax-4* mutants displayed an enhanced locomotion adjustment compared to wild type worms (p<0.01; Fig. 3C)—a striking difference from the lack of speed changes seen in *tax-2* mutants. Furthermore, *tax-4* mutants exhibited significantly increased basal locomotion rates in both chambers (Fig 3B), suggesting that alterations in basal locomotion do not interfere with experience-dependent movement adjustments.

Consistent with the idea that basal and experience-dependent locomotion are regulated independently, we found that wild type worms cultivated at either 23°C or 17°C and then transferred to 20°C during chamber exploration exhibited significantly changes in basal locomotion speed but had little corresponding effect on the extent of context-dependent modulation (Supp Fig. 2A). Specifically, temperature shifts were positively correlated with basal locomotion rates (binary chambers: *r* = 0.52, p < 0.0001; uniform chambers: *r* = 0.48, p < 0.0001; Suppl. Fig. 2B), yet no significant correlation was observed between temperature shifts and Δspeed (*r* = −0.03, p = 0.22; Suppl. Fig. 2C), reinforcing the idea that these two processes operate independently.

Together, these observations indicate that TAX-2 is critical for integrating prior experience into locomotion modulation, while TAX-4 primarily regulates basal locomotion. Despite both pathways engaging cGMP signaling, their functional independence suggests distinct regulatory mechanisms for basal locomotion and context-dependent adjustments.

Given that four other genes (*cng-1, cng-2, cng-3* and *cng-4*) are predicted to encode CNG channels in *C. elegans,* we investigated their role in locomotion modulation in microfluidic chambers [67]. Due to abnormal development in *cng-1* mutants, we were unable to assess their locomotion; however, we were able to evaluate mutants of the other three genes. We found that *cng-3(gk541751)* mutant worms failed to adjust locomotion speed in the microfluidic chambers, while *cng-2(tm4267)* and *cng-4(gk195496)* mutants exhibited normal modulation comparable to wild type worms (Fig. 3D). The identification of CNG-3 was particularly intriguing, as recent studies suggest that it mediates changes in AFD neuronal activity based on prior temperature experiences [68,69], indicating that CNG-3 contributes to neural plasticity rather than direct stimulus detection. Our results further support this view by showing that CNG-3 plays a broader role in locomotion modulation in environments lacking thermal gradients, which suggests its involvement in neuronal plasticity beyond the thermosensory modality.

Next, we determined whether AFD is the site where CNG-3 functions in context-dependent locomotion modulation. To do so, we introduced single-copy transgenes expressing *cng-3* cDNA under the control of two AFD-specific promoters, *srtx-1Bp* and *gcy-18p*, into *cng-3(gk541751)* mutant worms. Regardless of the promoter used, AFD-specific expression of *cng-3* restored the locomotion speed modulation (Δspeed: 47 ± 8% for *srtx-1Bp*::*cng-3* and 40 ± 6% for *gcy-18p::cng-3*) to levels similar to wild type (Fig 3E). These results demonstrate that AFD neurons are the site of action for CNG-3 in mediating context-dependent locomotion modulation. Overall, our findings elucidate an unexplored role for AFD beyond thermosensation, revealing that components of a cGMP signaling pathway enable AFD to modulate behavior in an experience-dependent manner.

### AFD sensory microvilli are dispensable for locomotion modulation, but AFD neurons are required.

**Figure 4.**
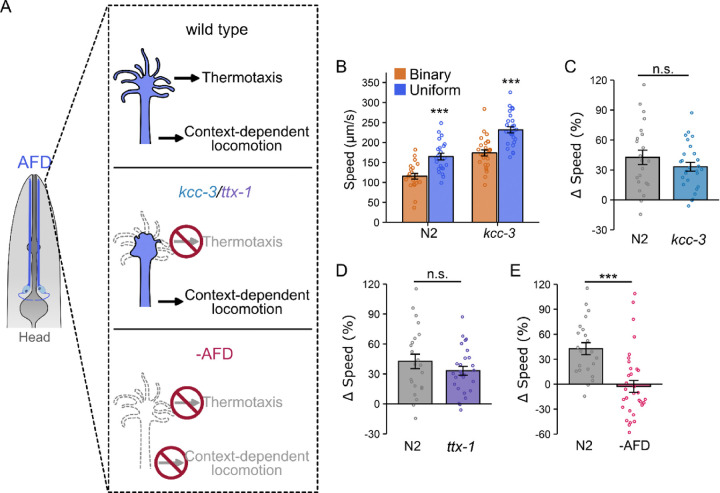
AFD, but not its sensory endings, is required for context-dependent locomotion modulation. (A) Schematic representation of AFD function in temperature sensing and locomotion modulation. AFD sensory endings are essential for thermosensation but dispensable for context-dependent locomotion modulation. (B) Locomotion speed (μm/s) of wild type N2 and *kcc-3* mutant worms in the binary (orange) and uniform (blue) chambers. The *kcc-3* mutants preserve context-dependent locomotion modulation while exhibiting increased basal locomotion rates. (C) Speed differences (Δspeed) for N2 and *kcc-3* mutant worms, and (D) for N2 and *ttx-1* mutant worms, respectively. Context-dependent locomotion modulation remains in *kcc-3* and *ttx-1* mutant worms, although they abolish the AFD thermosensory function. (E) Ablation of AFD eliminates the context-dependent locomotion modulation. Data are presented as mean ± SEM. Each data point represents the mean behavior of worms within a single chamber. Statistical significance was determined using an unpaired Student’s t-text (n.s. indicates not significant, and ***: p < 0.001).

The finding that cGMP signaling genes (such as *gcy-18* and *cng-3*) in AFD neurons modulate context-dependent locomotion prompted us to investigate whether the AFD thermosensory apparatus, crucial for temperature detection [36], is also necessary for locomotion modulation in our microfluidic assay. Previous studies have shown that the sensory microvilli at the tip of the AFD dendrite (Fig. 4A) are essential for temperature sensing and thermotaxis [36,38]. For example, mutations in the *kcc-3* gene, which encodes a K^+^/Cl^−^ cotransporter in the amphid sheath glia, disrupt microvilli formation and significantly impair thermotaxis towards their cultivation temperature [37]. Despite these thermotaxis defects, we found that context-dependent locomotion modulation remained unchanged in *kcc-3(ok228)* mutant worms (Δspeed: 33 ± 4%) compared to wild type (42 ± 7%; p = 0.28; Fig. 4B, C). Interestingly, *kcc-3* mutant worms displayed elevated basal locomotion rates in both chamber types (Fig. 4B), a phenomenon also observed in *tax-4* mutants. This further supports our earlier finding that alterations in basal locomotion do not correlate with context-dependent locomotion modulation (Suppl. Fig. 2B, C). Together, these findings suggest that the role of AFD neurons in modulating context-dependent locomotion is distinct from their thermosensory functions and differs from the mechanisms controlling basal locomotion.

To further confirm that the sensory endings of AFD are not required for context-dependent locomotion modulation, we examined *ttx-1(767)* mutant worms, which completely lack microvilli and exhibit more severe thermotaxis defects than those seen in *kcc-3* mutants [38]. Consistent with the notion that AFD has separate roles in context-dependent locomotion and thermosensation, *ttx-1* mutants still display locomotion modulation in microfluidic chambers. However, the extent of modulation in these mutants was slightly reduced and more variable (Δspeed: 29 ± 10%) compared to wild type animals (42 ± 7%, p = 0.29, Fig. 4D). It is worth noting that *ttx-1* mutations disrupt the expression of multiple AFD genes, including those involved in cGMP signaling [70], which may explain the increased variability in locomotion modulation. Nonetheless, despite completely lacking the AFD sensory apparatus, the ability of *ttx-1* mutant worms to adjust their locomotion speed further demonstrates that AFD neurons modulate context-dependent locomotion via mechanisms distinct from those used in thermosensation.

Surprised to find that the AFD sensory apparatus is dispensable for locomotion modulation, we next examined worms with genetically ablated AFD neurons. To achieve targeted AFD ablation, we used a published strain with an integrated transgene expressing caspase protein under the AFD-specific promoter *gcy-8p* [71]. AFD-ablated worms lost the ability to adjust locomotion rates in microfluidic chambers, as indicated by a significant reduction in Δspeed (wild type: 42 ± 7%, AFD ablated: −2 ± 7%, p <0.001; Fig. 4E). These results demonstrate that although the sensory endings of AFD are not required for locomotion adjustment, the AFD neuron itself is essential.

### Context-dependent locomotion modulation requires the MEC-10 mechanosensory channel subunit.

**Figure 5.**
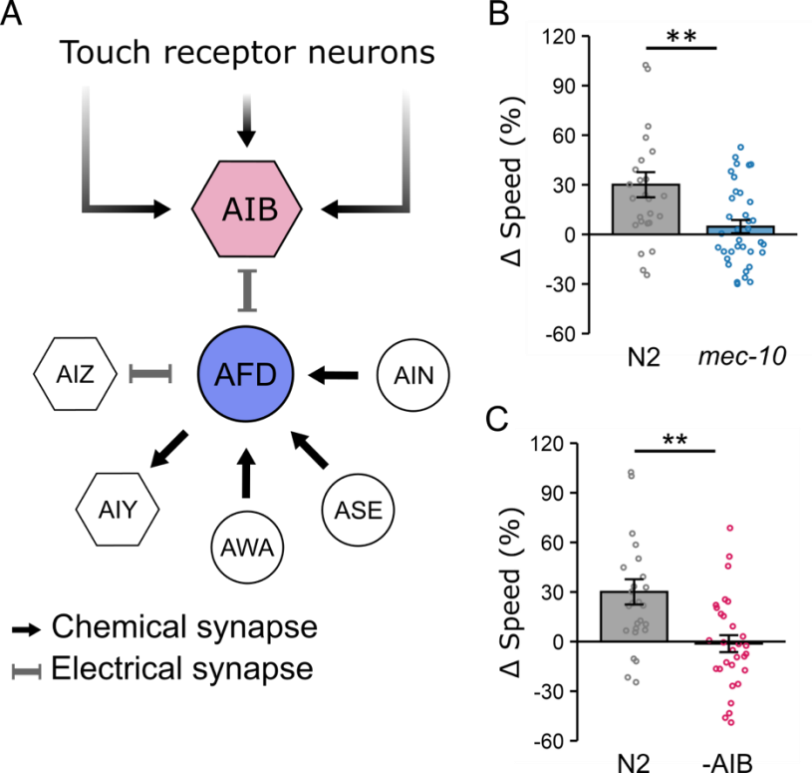
Context-dependent locomotion adjustments require both the mechanosensory channel subunit MEC-10 and the AIB interneurons that connect AFD to touch circuits. (A) Schematic representation of neurons synapsing with AFD. AIB provides the shortest pathway linking AFD to touch receptor neurons. (B, C) Speed differences (Δspeed) for N2, *mec-10* mutants, and AIB-ablated (-AIB) worms. Both *mec-10* mutants (B) and AIB-ablated worms (C) fail to modulate locomotion in microfluidic chambers. Data are presented as mean ± SEM. Each data point represents the mean behavior of worms within a single chamber. Statistical significance was determined using one-way ANOVA followed by a Tukey–Kramer post hoc test (**: p < 0.01).

We investigated whether mechanosensation is necessary for worms to perform context-dependent locomotion adjustments in microfluidic chambers. This was prompted by previous findings showing that touch sensation is critical for *C. elegans* to select their preferred space through interactions with physical structures [52]. The *mec-10* gene encodes an amiloride-sensitive Na^+^ channel protein required for sensing body touch. Mutations in *mec-10* disrupt mechanotransduction channels in the worms touch-receptor neurons (TRNs), impairing touch sensitivity [72,73]. We found that *mec-10(tm1552)* mutants failed to display locomotion adjustment (Δspeed: 5 ± 4%, Fig. 5B). These results show that *mec-10* is crucial for context-dependent locomotion modulation, supporting the hypothesis that locomotion is modulated depending on the worm’s tactile experience.

### AIB interneurons connecting TRNs and AFD are crucial for tactile-dependent behavioral plasticity.

Touch receptor neurons (TRNs) do not directly synapse onto AFD, suggesting that additional neurons are required to bridge them for tactile-dependent locomotion modulation. Examination of the *C. elegans* connectome revealed that AIB serves as the shortest network pathway connecting TRNs to AFD (Fig. 5A). This positions AIB as a key candidate for relaying signals from TRNs to AFD. If this is correct, we expect AIB to be necessary for tactile-dependent modulation. To examine this, we quantified the locomotion of AIB-ablated worms in microfluidic chambers. For this, we employed a previously published strain expressing caspase under an AIB-specific promoter (*npr-9p*), resulting in ablation of the AIB neuron pair [74]. We found AIB-ablated worms failed to adjust their speed in chambers, showing a significant reduction in Δspeed (−1 ± 5%) compared to wild type (32 ± 8%, p <0.01, Fig. 5C). These results demonstrate that the AIB interneurons are crucial for tactile-dependent locomotion modulation.

### Mutations in *innexin* genes disrupt tactile-dependent modulation.

**Figure 6.**
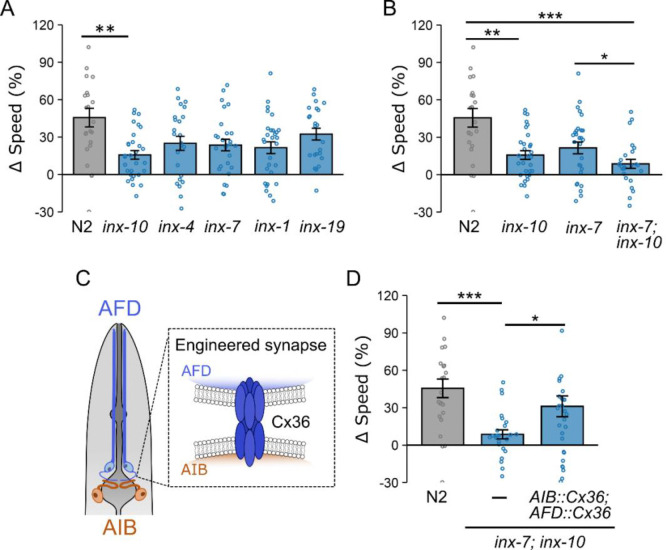
Tactile-dependent locomotion modulation is disrupted in mutant worms lacking gap junction genes and is restored by engineered Cx36 electrical synapses linking AFD and AIB. (A) Speed differences (Δspeed) for *inx-1, 4, 7, 10, 19* mutant worms. (B) Speed differences for *inx-7; inx-10* double mutants. (C) Schematic illustrating engineered electrical synapses formed by Cx36 when expressed in adjacent neurons. (D) Tactile-dependent locomotion modulation is restored in *inx-7; inx-10* double mutants by expressing Cx36 in AFD and AIB neurons. Data are presented as mean ± SEM. Each data point represents the mean behavior of worms within a single chamber. Statistical significance was determined using one-way ANOVA followed by a Tukey–Kramer post hoc test (*: p < 0.05; **: p < 0.01, and ***: p < 0.001).

Electrical synapses between AIB and AFD in *C. elegans* are formed by gap junction proteins called innexins [75]. If these AIB–AFD connections are important for tactile-dependent modulation, then mutations in innexin genes should impair locomotion adjustments in microfluidic chambers. To examine this hypothesis, we investigated loss-of-function mutants of various innexin genes. Because *C*. *elegans* has 25 innexin genes [76,77], we focused on those known to be expressed in AFD and AIB [78–80], identifying three genes (*inx-4, inx-10* and *inx-19)* expressed in AFD*,* one gene (*inx-1)* in AIB, and one gene (*inx-7*) in both*.* We found that *inx-10* mutants exhibited a partial but significant decrease in locomotion adjustments (Δspeed: 15 ± 3%) compared to wild type worms (45 ± 7%, p <0.001, Fig. 6A). Similarly, mutations in *inx-1, inx-4, and inx-7* all lowered Δspeed values, although these differences were not statistically significant relative to wild type worms (Fig. 6A). Furthermore, we found that *inx-7; inx-10* double mutants exhibit a lower Δspeed compared to *inx-7* or *inx-10* single mutants (Fig. 6B). These findings suggest that disrupting innexins in AFD and AIB impair tactile-dependent locomotion modulation.

### Re-establishing AIB-AFD electrical synapses restores tactile-dependent locomotion modulation in *inx-7; inx-10* double mutants.

If the tactile-dependent modulation defects in innexin mutants result from disrupted AIB-AFD electrical synapses, then reintroducing a synthetic gap junction specifically between these neurons should rescue locomotion modulation. To test this, we employed a recently developed approach that introduces engineered electrical synapses by expressing the mammalian gap junction protein Cx36 in *C. elegans* neurons. Several studies have shown that engineered Cx36 synapses in *C. elegans* facilitate electrical coupling between neurons, support behavioral modulation, and circumvent chemosensory impairments in damaged neural circuits [81–84]. Based on these findings, we introduced Cx36 into AFD and AIB neurons in *inx-7; inx-10* double mutants (Fig. 6C). Remarkably, expression of Cx36 in both AFD and AIB significantly improved locomotion adjustments in *inx-7; inx-10* mutants, increasing Δspeed from 15 ± 3% to 31 ± 8% (p <0.05, Fig. 6D). These findings demonstrate that engineered electrical synapses between AFD and AIB can restore tactile-dependent locomotion modulation.

## DISCUSSION

Our study reveals that the thermosensory AFD neuron plays an unexpected role in coupling tactile experiences to locomotion modulation in *C. elegans*. We show that: 1) *C. elegans* exhibit distinct locomotion patterns in identical environments, influenced by prior tactile experiences; 2) tactile-dependent behavior modulation requires the AFD neuron but not its thermosensory dendritic endings; 3) thermotaxis and tactile-dependent locomotion modulation rely on distinct sets of cGMP signaling proteins and CNG channel subunits; 4) the AIB interneuron, which connects AFD to mechanosensory circuits, is essential for tactile modulation; and 5) electrical synapses between AFD and AIB are critical for this process. Below, we discuss the implications of these findings.

### A thermosensation-independent role for AFD in tactile-dependent behavior modulation.

Our findings highlight the remarkable versatility of sensory neurons in shaping behaviors beyond their primary functions. This supports the emerging view that sensory neurons have multifaceted roles in circuit modulation, extending well beyond canonical sensory activities. Such complexity is encoded at multiple levels within neural circuits. In *C. elegans*, for instance, the ASH neuron drives avoidance responses to various noxious stimuli, including hyperosmolarity, nose touch, and volatile repellents, demonstrating polymodal functionality [85–88]. Similarly, the olfactory receptor neuron AWC exhibits stochastic responses to temperature, introducing variability that enhances the flexibility and reliability of thermotactic behavior [89–91]. In contrast to these examples, the role of AFD in tactile-dependent plasticity does not rely on its sensory endings but is mediated through synaptic connectivity. This reveals an unexpected mechanism by which AFD influences context-dependent behavioral plasticity via network-level interactions rather than direct sensory activity.

### Distinct cGMP signaling pathways in AFD mediate sensation and plasticity.

Our data indicates that the CNG channel α subunits TAX-4 and CNG-3 play distinct roles in AFD function. Previous work has shown that TAX-4 is essential for AFD to detect temperature and generate thermally evoked responses [40], whereas CNG-3, although dispensable for temperature sensing, is crucial for tactile-dependent locomotion modulation. Supporting a broader role in sensory plasticity, CNG-3 is required in AWC neurons for short-term odor adaptation [92] and in AFD for adjusting temperature thresholds based on prior thermal experiences [68]. Our demonstration of the essential role of CNG-3 in tactile-dependent locomotion modulation further suggests that CNG-3 enables context-dependent behavioral plasticity rather than directly mediating stimulus detection.

CNG channels are heteromeric complexes composed of both α and β subunits. Our data, along with previous work, show that TAX-2, the CNG β subunit in *C. elegans*, is required for both tactile-dependent locomotion modulation and thermotransduction [40]. This finding implies that TAX-2 can partner with different α subunits to support sensory detection and neural plasticity. This functional specialization is reminiscent of photoreceptor neurons, where the composition and ratio of CNG channel subunits determine light sensitivity and enable contrast detection under varying light intensities [93–95].

In addition to the diversity of CNG channels, guanylyl cyclases in AFD also exhibit functional specificity. For example, mutations in *gcy-18* alone significantly disrupt tactile-dependent locomotion modulation without substantially affecting temperature sensing [41,43], whereas *gcy-8* is essential for generating thermally evoked Ca^2+^ responses [41,42] yet appears dispensable for tactile-dependent plasticity. Together, these results show that AFD uses distinct sets of guanylyl cyclases and CNG channels to execute dual roles—precise temperature sensing and context-dependent behavioral modulation.

### Differential regulation of context-dependent plasticity and basal locomotion rates in AFD.

Our results further suggest that the mechanisms controlling basal locomotion and context-dependent behavioral modulation are not only distinct but also operate independently. Temperature fluctuations significantly affect basal locomotion rates, i.e., higher temperatures accelerate speed, while lower temperatures slow it down [96,97]. However, tactile-dependent locomotion modulation in microfluidic chambers remains robust despite these temperature-induced changes in basal movement. This independence is further supported by our observations in *tax-4* and *kcc-3* mutants, which display elevated basal locomotion rates yet still adjust their locomotion in response to tactile experiences. These findings indicate that the processes governing basal locomotion and context-dependent modulation are controlled by separate, non-interfering mechanisms, even though both pathways engage cGMP signaling.

### Electrical synapses bridge distinct circuits for behavioral modulation.

AIB-AFD electrical synapses are pivotal for tactile-dependent behavioral plasticity, serving to connect mechanosensory and thermosensory circuits. Our results show that disrupting the electrical connection between AFD and AIB impairs tactile-dependent locomotion modulation, whereas reintroducing engineered synapses restores it. The successful replacement of invertebrate innexins with vertebrate connexins, proteins that perform similar functions despite lacking sequence homology, indicates that the core function of these synapses lies in their connectivity rather than in their specific protein sequences. Previous models have proposed that AIB-AFD electrical synapses contribute to avoidance behavior by forming part of an FLP-AIB-AFD circuit, with AIB serving as a hub neuron that integrates sensory inputs via chemical synapse with FLP and electrical synapse with AFD [98]. Our experiments using engineered synapses elucidate the functional importance of the AIB-AFD connection in tactile-dependent locomotion modulation. Moreover, the transfer of signaling molecules such as Ca^2+^ and cGMP through electrical synapses likely facilitates the coordination of neuronal activity across distinct networks [99–102]. Although the precise mechanisms require further investigation, our study demonstrates that AIB-AFD electrical synapses are essential for incorporating tactile context into locomotion modulation.

**IN SUMMARY**, our findings reveal an unexpected role for AFD in tactile-dependent behavioral plasticity. They show that specialized molecular mechanisms (including distinct cGMP signaling pathways, diverse guanylyl cyclases, and functional electrical synapse connectivity) enable AFD sensory neurons to perform dual functions: detect stimuli and support context-dependent plasticity. This work expands our understanding of sensory neuron versatility supporting adaptive behavior in animals.

## METHODS

### Nematode Strains and Growth –

*C. elegans* strains were maintained under standard conditions at 20°C on nematode growth medium (NGM) agar plates [103]. These plates were seeded with *E. coli* OP50 lawns. Strains used in this study are listed in Table 1.

### Single Copy Insertion of Transgenes –

*Mos1*-mediated transgene insertion methods were used to generate transgenic animals carrying single-copy transgenes [104,105]. Briefly, worms carrying the *ttTi5605 Mos1* site on chromosome II were injected with a mixture of plasmids encoding the transgene of interest (40 ng/μl) and Cas9 protein plus guide RNA specific to the *Mos1* site (60 ng/μl). To aid in selection of successful insertions, the inserted transgene also included markers *vha-9p::mScarlet* and *rps-0p::hygR*, which confer red fluorescence in the gut and hygromycin resistance, respectively.

### Transgenes and Germline Transformation –

Strains carrying extrachromosomal arrays were generated by microinjecting various plasmids together with co-injection markers. The co-injection markers included *unc-112p::gfp* (coelomocyte, 30 ng/μl), *rpl-28p::neoR* (20 ng/μl), and *Cbr-unc-119* (20 ng/μl). The blank vector pBluescript was used as filler DNA to bring the final total DNA concentration to 100 ng/μl. A detailed list of the plasmids used to create transgenic worms in this study is provided in Table 2.

### Fabrication of PDMS Microfluidic Chambers –

Microfluidic chambers were designed in AutoCAD (S1. File), and the resulting CAD designs were used to fabricate SU8-Silicon molds with a height of 100 μm using photolithography (FlowJem, NO, Canada). These molds were then used to fabricate microfluidic chambers with various physical features using soft lithography with polydimethyl siloxane (PDMS, Sylgard 184, Dow Corning, MI). Specifically, PDMS prepolymer was poured onto the SU8-silicon molds and cured at 80°C for 2 hours. Once cured, the PDMS layer was carefully lifted away from the silicon wafer, and a 2 mm skin biopsy punch (Acuderm Inc., FL) was used to create inlet and outlet holes. To assemble the device, the PDMS layer was place onto a microscope slide (1 mm thick), and the assembly was treated in a plasma cleaner (PDC-32G, Harrick Scientific, NY) for 1 minute. Distilled water was added to the plasma-treated devices within 5 minutes. Before loading the worms, the devices were thoroughly rinsed with M9 buffer.

### Behavior Analysis –

Worms were age-synchronized for behavioral assays by transferring 40–50 young adult worms onto an NGM plate seeded with OP50 and allowing them to lay eggs for 2 hours before removal. The plates were then kept at room temperature (20 ± 2°C) for 72 hours, allowing the eggs to develop into young adults. To wash the synchronized adults off the plate, worms were gently transferred into Eppendorf tubes using glass Pasteur pipettes. After allowing the worms to settle at the bottom of the tube (~1 minute), the supernatant was removed and replaced with fresh M9 buffer. This washing step was repeated three times to remove residual OP50. Next, approximately 30–70 worms were loaded into the chamber inlet using a 20μl pipette and allowed to explore the chamber freely for 60 minutes before recording. Videos were captured for 10 seconds at 7.5 frames per second using a Basler acA2440 camera (Basler, PA) equipped with an AF Micro-NIKKOR 60 mm lens (Nikon, NY). Behavioral data were extracted from the videos using WormLab software (MBF Bioscience, VT) through individual worm tracking. Only those worms that were already within the assay area at the start of the recording were analyzed, and any worms that entered later were excluded. Crawling speed was determined by the total distance traveled by a worm during the 10 second recording. A turn was defined as a change in crawling direction of 15° or more. Reversals were defined as sudden movement in the opposite direction for at least 5 frames (~0.6 seconds). Worms were considered idle if their absolute velocity (distance traveled between frames) was below 20 μm/s for more than 10 frames (~1.3 seconds).

### Statistical Analysis –

Behavior measurements were calculated for each individual chamber, and these chamber-specific values were then used to generate the final mean values shown in the bar plots. The sample size was defined as the number (approximately 25) of chambers analyzed per experiment. An unpaired Student’s t-test with Bonferroni correction was used to compare average speeds between uniform and binary chambers. To quantify the relative difference between groups, we calculated the percent change for each value in the uniform chambers relative to the mean of value from the binary chambers. Specifically, we subtracted the mean of the values from the binary chamber from each individual value in the uniform chamber. The result was then divided by the mean of the binary chamber and multiplied by 100 to express the difference as a percentage. For comparisons among multiple strains, we conducted one-way ANOVA followed by a Tukey–Kramer post hoc test on all possible pairwise comparisons. All statistical analyses were performed using R.

## Supplementary Material

Supplement 1

## Figures and Tables

**Table 1.** List of *C. elegans* strains used in this study

Strain	Genotype	Citation
PR694	*tax-2(p694) I*	[106]
PR678	*tax-4(p678) III*	[65]
FX31482	*cng-2(tm4267) IV*	[107]
VC40261	*cng-3(gk541751) IV*	[108]
VC20290	*cng-4(gk195496) IV*	[108]
IK800	*gcy-8(oy44) IV.*	[43]
VC30137	*gcy-18(gk423024) IV*	[108]
VC20451	*gcy-12(gk142661) II*	[108]
BJH3481	*gcy-18(gk423024) IV, pekSi609[gcy-18p::gcy-18(cDNA)::sl2::mNeonGreen::unc-54 3’utr, vha-6p::mScarlet, hygR(+)] ttTi5605 II*	This study
BJH3527	*pekSi655[srtx-1Bp::cng-3(cDNA)::sl2::mNeonGreen::unc-54 3’utr, vha-9p::mScarlet, hygR(+)] ttTi5605 II; cng-3(gk541751) IV*	This study
BJH3537	*tax-2(p694) I; pekSi665[srtx-1Bp::tax2(cDNA)::sl2::mNeonGreen::unc-54 3’utr, vha-9p::mScarlet, hygR(+)] ttTi5605 II*	This study
BJH3539	*pekSi667 [gcy-18p::cng-3(cDNA)::sl2::mNeonGreen::unc-54 3’utr, vha-9p::mScarlet, hygR(+)] ttTi5605 II; cng-3(gk541751) IV*	This study
GN112	*pgIs2[gcy-8p::TU#813, gcy-8p::TU#814, unc-122p::GFP, gcy- 8p::mCherry, gcy-8p::gfp, ttx-3p::gfp]*	[71]
LX1024	*kcc-3(ok228) II*	[37]
PR767	*ttx-1(p767) V*	[38]
JN578	*peIs578 [npr-9p::casp1 + npr-9p::venus + unc-122p::mCherry]* (genetically ablated AIB)	[74]
ZB2551	*mec-10(tm1552) X*	[72]
CB1611	*mec-4(e1611) X*	[109]
FG927	*inx-1(tm6662) X.*	[110]
FG0614	*inx-4 (ok2373) V*	[80]
RB1792	*inx-7(ok2319) IV*	[110]
RB2051	*inx-10(ok2714) V*	[110]
FX01896	*inx-19(tm1896) I*	[111]
BJH2919	*inx-7(ok2319) IV; inx-10(ok2714) V*	This study
BJH1162	*pekEx302 [srtx-1Bp::cx36::sl2::mNeonGreen, inx-1p::cx36::sl2::mKate, Cbr-unc-119(+), unc-122p::GFP, neoR(+)]; unc-119(ed3) III; inx-7(ok2319) IV; inx-10(ok2714) V*	This study

**Table 2.** List of plasmids used to create transgenic *C. elegans* strains

Name	Contruct	Notes
BJP-MR16	*gcy-18p::gcy-18(mini-gene)::sl2::mNeonGreen::rab-3 3’utr; vha-9p::mScarlet::tbb-2 3’utr; rps-0p::hygR*	The *gcy-18* mini-gene includes all exons and introns 11, 12, and 13.
BJP-MR65	*srtx-1Bp::cng-3(cDNA)::sl2::mNeonGreen::rab-3 3’utr; vha-9p::mScarlet::tbb-2 3’utr; rps-0p::hygR*	
BJP-MR72	*srtx-1Bp::tax2(cDNA)::sl2::mNeonGreen::rab-3 3’utr; vha-9p::mScarlet::tbb-2 3’utr; rps-0p::hygR*	
BJP-MR93	*gcy-18p::cng-3(cDNA)::sl2::mNeonGreen::rab-3 3’utr; vha-9p::mScarlet::tbb-2 3’utr; rps-0p::hygR*	
BJP-MR96	*inx-1p::cx36::sl2::mKate::rab-3 3’utr*	
BJP-MR97	*srtx-1bp::cx36::sl2::mKate::rab-3 3’utr*	
