# Supplementary material for: AFD Thermosensory Neurons Mediate Tactile-Dependent Locomotion Modulation in C. elegans
Source: bioRxiv. 2025 Feb 24:2025.02.19.639001. Preprint. [Version 1] doi: 10.1101/2025.02.19.639001 (PMC11888201; doi:10.1101/2025.02.19.639001)
Supplement: Supplement 1 [file NIHPP2025.02.19.639001v1-supplement-1.pdf]

bioRxiv preprint doi: https://doi.org/10.1101/2025.02.19.639001; this version posted February 24, 2025. The copyright holder for this preprint (which was not certified by peer review) is the author/funder. This article is a US Government work. It is not subject to copyright under 17 USC 105 and is also made available for use under a CC0 license.

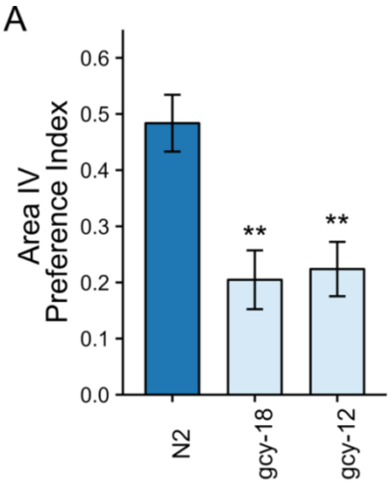

Suppl. Figure 1. Worms lacking either *gcy-18* or *gcy-12* failed to identify their preferred area in microfluidic chambers. Preference index values of wild type N2 worms, *gcy-18* mutants, and *gcy-12* mutants were determined as described by Han *et al.* [52]. Wild type worms exhibit a strong preference for a specific zone (zone IV, as defined by Han *et al*. [52]), whereas both *gcy-18* and *gcy-12* mutants show significantly reduced preference compared to wild type worms. Bars represent mean ± SEM. Statistical comparisons were performed using one-way ANOVA followed by a Dunnett's test for multiple comparisons (**p < 0.01).

bioRxiv preprint doi: https://doi.org/10.1101/2025.02.19.639001; this version posted February 24, 2025. The copyright holder for this preprint (which was not certified by peer review) is the author/funder. This article is a US Government work. It is not subject to copyright under 17 USC 105 and is also made available for use under a CC0 license.

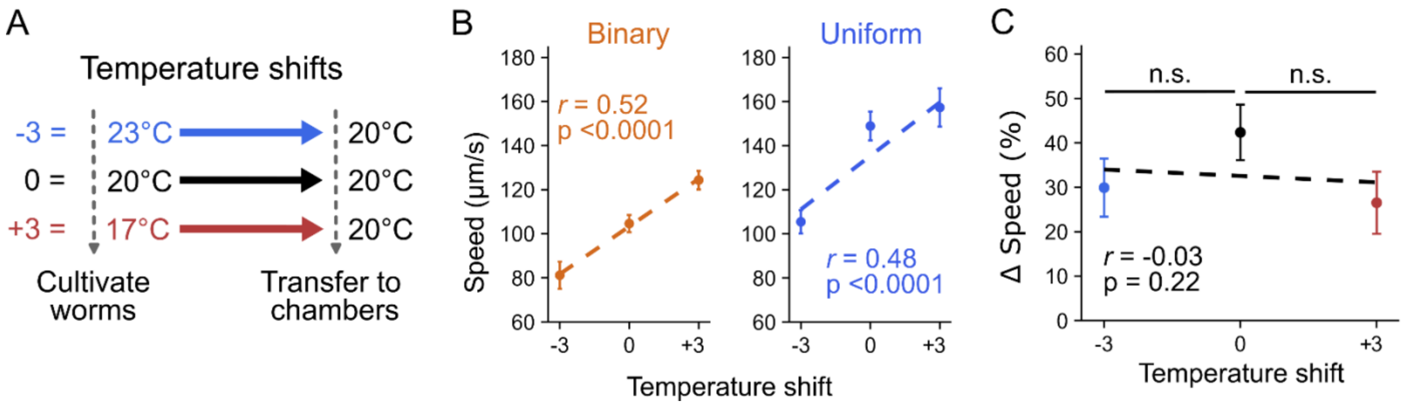

Suppl. figure 2. Temperature shifts do not affect tactile-dependent modulation. (A) Schematic of experimental design: Worms were reared at three different temperatures (17°C, 20°C, and 23°C) and then transferred to 20°C for 1 hour during the behavioral assay. (B) Locomotion speed of wild type N2 worms in the binary (orange) and uniform (blue) chambers after experiencing temperature shifts. Linear regression analysis reveals a significant positive correlation between rearing temperature and locomotion speed in both chambers. (C) Differences in speed (∆speed) between worms experiencing different temperature shifts. Linear regression significance test shows no significant relationship between ∆speed and temperature shift. Points represent mean ± SEM.
